# Supplementary material for: Heat adaptation of phage T7 under an extended genetic code
Source: Virus Evol. 2021 Dec 1;7(2):veab100. doi: 10.1093/ve/veab100 (PMC8923235; doi:10.1093/ve/veab100)
Supplement: veab100_Supp [file veab100_supp.zip › Sequencing_Analysis.zip!/Sequencing_Analysis/A1/evidence/JC_SIDE_1_55.html]

BRESEQ :: Evidence


| New junction evidence | | | | | | | | | | | |
| --- | --- | --- | --- | --- | --- | --- | --- | --- | --- | --- | --- |
|  | | seq id | position | reads (cov) | reads (cov) | score | skew | freq | annotation | gene | product |
| \* | ? | NC\_001604.1 | = 13244 | 2141 (0.690) | 49 (0.020) | 39/582 | 27.3 | 4.5% | intergenic (–/–) | *–/–* | –/– |
|  | ? | NC\_001604.1 | 14067 = | 6 (0.000) |  |  |  |  | intergenic (–/–) | *–/–* | –/– |
| Rejected: Coverage evenness skew score above cutoff. | | | | | | | | | | | |
| Rejected: Frequency below/above cutoff threshold. | | | | | | | | | | | |

Only 100 of 2847 total aligned reads displayed.  

`GGTCGTAATTTGTCACCTTAAGAACCCAGACAAAGGTAAAGCACATGAGGAAGGTCGCCCCGTTTCTATTACTGACCTACGTGGTTCTGGCGCACTACGCCAACTATCTGATACTATTATTGCCCTTGAGCGTAATCAGCAAGGCGATATGCCTAACCTTGTCCTCGTTCGTATTCTCAAGTGCCGCTTTACTGGTGATACTGGTATCGCTGGCTACATGGAATACAACAAGGAAACCGGATGGCTTGAACCATCAAGTTACTCAGGGGAAGAAGAGTCACACTCAGAGTCAACAGACTGGTCCAACGACACTGACTTCTGACAGGATTCTTGATGACTTTCCAGACGACTACGAGAAGTTTCGCTGGAGAGTCCCATTCTAATACGACTCACTAAAGGAGACACACCATGTTCAAACTGATTAAGAAGTTAGGCCAACTGCTGGTTCGTATGTACAACGTGGAAGCCAAGCGACTGAACGATGAGGCTCGTAAAGAGGCCACACAGTCACGCGCTCTGGCGATTCGCTCCAACGAACTGGCTGACAGTGCATCCACTAAAGTTACCGAGGCTGCCCGTGTGGCAAACCAAGCTCAACAGC  >  NC_001604.1/12944‑13544`  

                                                                                                                                                                                                                                                                                                            `|`                                                                                                                                                                                                                                                                                                              
       `aTTTGTCACCTTAAGAACCCAGACAAAGGTAAAGCACATGAGGAAGGTCGCCCCGTTTCTATTACTGCCCTACGTGGTTCTGGCGCACTACGCCAACTATCTGATACTATTATTGCCCTTGAGCGTAATCAGCAAGTCGATATGCCTAACCTTATCCTCGTTCGTATTCTCAAGTGCCGCTTTACTGGTGATTCTGGTATCGCTGGCTACATGGAATACAACAAGGAAACCGGATTGCTTGACCCATCAAGTTACTCAGGGGAAGAAGATTCACACTCCGAGTCAACAGACCTGTcccttt                                                                                                                                                                                                                                                                                                       >  1:39821/1‑297 (MQ=255)`  
                  `tataaaCCCAAAAAATGTTAAAGACCGTTAGGAAGCTCGCCTCGTTTATATTACTGACCTACGTGGTTCTGGCGCACTACGCCAACTATCTGTTACTTTTATTGCCCTTGAGCTTCATCAGCCACGCGATATGCCTAACCTTGTCCTTGTTCGTATTTTCAAGTGCCGCTTTACTGGTGATACTGGTATCGCTGGCTACATGGAATACAACAAGGAAACCGGATGGCTTGAACCATCAAGTTACTCAGGGGAAGAAGAGTCACACTCAGAGTCAACAGACTGGTCCAACGACACTGACTTc                                                                                                                                                                                                                                                                                            <  1:456278/297‑1 (MQ=255)`  
                            `gACAAAGGTAAAGCACATGAGGAAGGTCGCCCCGTTTCTATTACTGACCTACGTGGTTCTGGCGCACTACGCCAACTATCTGATACTATTATTGCCCTTGGGCGTAATCAGCAAGGCGATATGCCTAACATTGTCCTCGTTCGTATTCTCAAGTGCCGCTTTCCTGGTGATACTGGTATCGCTGGCTACATGGAATACAACAAGGAAACCGGATGGCTTGAACCATCAAGTTACTACGGGGAAAAAGAGTCACACTCAGAGTCAACAGACTGGTCAAACGAAACTGACtctgtctcttata                                                                                                                                                                                                                                                                                  >  1:48994/1‑289 (MQ=255)`  
                                  `ggTAAAGCACATGAGGAAGGTCGCCCCGTTTCTATTACTGACCTACGTGGTTCTGGCGCACTACGCCAACTATCTGATACTATTATTGCCCTTGAGCGTAATCAGCAAGGCGATATGCCTAACCTTGTCCTCGTTCGTATTCTCAAGTGCCGCTTTACTGGTGATACTGGTATCGCTGGCTACATGGAATACAACAAGGAAACCGGATGGCTTGAACCATCAAGTTACTCAGGGGCAGAAGAGTCACACTCAGAGTCAACAGACTGGTCCAACGACACTGACTTCTGACAGGATTCTtgat                                                                                                                                                                                                                                                                            >  1:159316/1‑301 (MQ=255)`  
                                    `taagagacaGGAGTAAGGTCGCCCCGTTTCTATTACTGACCTACGTGGTTCGGGCGCACTACGCCAACTATCTGATACTATTAGTGCACTTGAGCATAATCAGCAAGGCGATATGCCTAACCTTGTCCTCGTTCGTATTCTCAAGTGCCGCTGTACTGGTGATACTGGTATCGCTGGCTACATGGAATACAACAAGGAAACCGTATGGCTTGAACCATCAAGTTACTCAGGGGAAGAAGAGTCACACTCAGAGTCAACAGACTGGTCCAACGACACTGACTTCTGACAGTATTCTtgatga                                                                                                                                                                                                                                                                          <  1:122418/295‑1 (MQ=255)`  
                                     `aaaGCACATGAGCAAGGTCGCCCCGTTTCTATTACTGACCTACGTGGTTCTGGCGCACTACGCCAACTATCTCATACTATTATTTCCCTGGAGCGTCATCAGCAAGGCGATATGCCTAACCTTGTCATCGTTCGTATTCTCAAGTGCCGCTTTACTGGTGATACTGGTGTCGCTGGCTACATGGAATACAACAAGGAATCCGGATGGCTTGCACCATCAAGTTACTCAGGGGAAGAAGAGTCACACTCAGAGTCAACAGACTGGTCCAACGACACTGACTTCTGACAGGATTCTTGATGAc                                                                                                                                                                                                                                                                         <  1:88800/301‑1 (MQ=255)`  
                                              `gAGGAAGGTCGCCCCGTTTCTATTACTGACCTACGTGGTTCTGGCGCACTACGCCAACTATCTGATACTATTATTGCCCTTGAGCGTAATCAGTAAGGCGATATGCCTAACCTTGTCCTCGTTCGTATTCTCAAGTGCCGCTTTACTGGTGATACTGGTATCGCTGGCTACATGGACTACAACCAGGAAACCGGATGGCTTGAACCATCAAGTTACTCAGGGGAAGAAGAGTCACACCCAGAGTCAACAGACTGGTCCAACGAAACTGACTTCTGACAGGATTCTTGATGACTTTCCAgac                                                                                                                                                                                                                                                                >  1:295635/1‑301 (MQ=255)`  
                                                           `ccGTTTCTATTACTGACCTGCATGGGTCTGGCGCACGACGCCAACGAGCGGATACGATTATTGCCCTTGAGCGTAATCAGCAAGGCGAGATGCCTAACCGTGTCCTCGTTCGTATGCTCAAGTGCCGCTTTACTGGTGATACGGGTAGCGCTGGCTACATGGAATACAACAAGGAAACCGGATGGCTTGAACCATCAAGTTACTCAGGGGAAGAAGAGTCACACTCAGAGTCAACAGACTGGTCCAACTACACTGACTTCTGACAGGATTCTTGATGACTTTCCAGACGACTACGAGAAGt                                                                                                                                                                                                                                                   <  1:230638/301‑1 (MQ=255)`  
                                                               `ttCTATTACTGACCTACGTGGTTCTAGCGAACTACGCAAACTATCTGATAATATTATTGACCTTGGGCGTAATACGCAAGGCGATCTGCCTAACCTTGTCCTCGTTCGTATTCACAAGTGCCGCTTTACTGGTGATACTGGTATCGCTGGCTATATGGATTACAAAAAGGAAACAGGATGGCTTGAACCATCAAGTTACTCAGGGGAAGAAGAGTCACACTCAGAGTCAACAGAATGGTACAACGAAACAGACGTCTGACAGGATTCTtgatgaactgtctcttatacacatctccacgcc                                                                                                                                                                                                                                               >  1:43918/1‑274 (MQ=255)`  
                                                                     `tACTGACCTACGTGGTTCTGGCGCACTACGCCAACTATCTGATACTATTATTGCCCTTGAGCGTAATCAGCAAGGCGATATGCCTAACCTTGTCCTCGTTCGTATTCTCAAGTTCCGCTTTACTGGTGATACTGGTATCGCTGGCTACATGGAATACAACAAGGAAACCGGATGGCTTGAACCATCAAGTTACTCAGGGGAAGAAGAGTCACACTCAGAGTCAACAGACTGGTCCAACGACACTGACTTCTGACAGGATTCTTGATGACTTTCCAGACGACTACGAGAAGTTTCGCTGgag                                                                                                                                                                                                                                         <  1:177888/301‑1 (MQ=255)`  
                                                                         `gACCTACGTGGTTCTGGCGCACTACGCCAACTATCTGATACTATTATTGCCCTTGAGCGTAATCAGCAAGGCGATATGCCTAACCTTGTCCTCGTTCGTATTCTCAAGTGCCGCTTTACTGGTGATACTGGTATCGCTGGCTACATGGAATACAACAAGGAACCCGGATGGCTTAACCCATCAAGTTACTCAGGGGAAGAAGAGTCACACTCAGAGTCAACAGACTTGTCCAACGACACTGACTTCTGACAGGATTCTTGATGACTTTCCAGACGACGACGAGAAGTTTCGCTGGCGAGTc                                                                                                                                                                                                                                     >  1:146493/1‑301 (MQ=255)`  
                                                                           `ccTATGTGGTTCTGGCGAACTACGCCAACTATCTGATACTATTATTGCCCTTGAGCGTAATCAGCAAGGCGATATGCCTACCCTTGTCCTCGTTCGTATTCTCAAGTGCCGCTTTACTGGTGATACTGGTATCGCTGGCTACATTGAATACAACAAGGAAACCGGATGGCTTGCACCATCAAGTTACTCAGTGGAAGCAGAGTCACACTCAGAGTCAACAGACTGGTCCAACGACACTGACTTCTGACAGGATTCTTGATGCCTTTCCAgactgtctcttatacccatctccgagcccacg                                                                                                                                                                                                                                   >  1:183087/1‑272 (MQ=255)`  
                                                                           `ccTACGTGGTTCTGGCGCACTACGCCAACTATCTGATACTATTATTGCCCTTGAGCGTAATCAGCAAGGCGATATGCCTAACCTTGTCCTCGTTCGTATTCTCAAGTGCCGCTTTACTGGTGATACTGGTATCGCTGGCTACATGGAATACAACAAGGAAACCGGATGGCTTGCACCATCAAGTTACTCAGGGGAAGAAGAGTCACACTCAGAGTCAACAGACTGTTCCAACGACACTGACTTCTGACCGGCTTCTTGATGACTTTCCAGACGCCTTCGAGAAGTTTCGCTGGAGCGTccc                                                                                                                                                                                                                                   >  1:435943/1‑301 (MQ=255)`  
                                                                               `gtgtataagagacagcTACGCCAACTATCTGATACTATTATTGCCCTTGAGCGTAATCAGCAAGGCGATATGCCTAACCTTGTCCTCGTTCGTATTCTCAAGTGCCGCTTTACTGGTGATACTGGTATCGCTGGCTACATGGAATACAACAAGGAAACCGGATGGCTTGAACCATCAAGTTACTCAGGGGAAGAAGAGTCACACTCAGAGTCAACAGACTGGTCCAACGACACTGACTTCTGACAGGATTCTTGATGACTTTCCAGACGACTACGAGAAGTTTCGCTGGAGAGTCCCATTc                                                                                                                                                                                                                               <  1:27696/286‑1 (MQ=255)`  
                                                                               `cGTGGTTCTGGCGCACTACGCCAACTATCTGATACTATTATTGCCCTTGAGCGTAATCAGCAAGGCGATATGCCTAACCTTGTCCTCGTTCGTATTCTCAAGTGCCGCTTTACTGGTGATACTGGTATCGCTGGCTACATGGAATACAACAAGGAAACCGGATGGCTTGAACCATCAAGTTACTCAGGGGAAGAAGAGTCACACTCAGAGTCAACAGACTGGTCCAACGACACTGACTTCTGACAGGATTCTTGATGACTTTCCAGACGACTACGAGAAGTTTCGCTGGAGAGTCCCATTc                                                                                                                                                                                                                               <  1:477805/301‑1 (MQ=255)`  
                                                                                 `tGGTTCTGTCGCACTACGCCAACTATCTGATACTATTATTGCCCTTGAGCGTAATCAGCAAGGCGATATGCCTAACCTTGTCCTCGTTCGTATTCTCAAGTGCCGCTTTACTGGTGATACTGGTATCGCTGGCTACATGGAATACAACAAGGAAACCGGATGGCTTGAACCATCAAGTTACTCAGGGGAAGAAGAGTCACACTCAGAGTCAACAGACTGGTCCAACGACACTGACTTCTGACAGGATTCTTGAcagttgtttcatatgaagagattgttaagtcacgataatcaataggag                                                                                                                                                                                                                             <  1:114572‑M1/301‑49 (MQ=255)`  
                                                                                   `gTTCTGGCGCACTACGCCAACTATCTGATACTATTATTGCCCTTGAGCGTAATCAGCAAGGCGATATGCCTAACCTTGTCCTCGTTCGTATTCTCAAGTGCCGCTTTACTGGTGATACTGGTATCGCTGGCTACATGGAATACAACAAGGAAACCGGATGGCTTGAACCATCAAGTTACTCAGGGGAAGAAGAGTCACACTCAGAGTCAACAGACTGGTCCAACGACACTGACTTCTGACAGGATTCTTGATGACTTTCCAGACGACTACGCGAAGTTTCGCTggctgtctcttatacaca                                                                                                                                                                                                                           >  1:150981/1‑285 (MQ=255)`  
                                                                                       `gacaggaCTACGCCAACTATCTGATACTATTATTGCCCTTGAGCGTAATCAGCAAGGCGATATGCCTAACCTTGTCCTCGTTCGTATTCTCAAGTGCCGCTTTACTGGTGATACTGGTATCGCTGGCTACATGGAATACAACAAGGAAACCGGATGGCTTGAAACATCAAGTTACTCAGGGGAAGAAGAGTCACACTCAGAGTCAACAGACTGGTCCAACGACACTGACTTCTTACAGGATTCTTGATGACTTTCCAGACGACTACGAGAAGTTTCGCTGGAGAGTCCCATTCTAATACGa                                                                                                                                                                                                                       <  1:258635/295‑1 (MQ=255)`  
                                                                                        `ggCGCACTACGCCAACTATCTGATACTATTATTGCCCTTGAGCGTAATCAGCAAGGCGATATGCCTAACCTTGTCCTCGTTCGTATTCTCAAGTGCCGCTTTACTGGTGATACTGGTATCGCTGGCTACATGGAATACAACAAGGAAACTGGATGGCTTGAACCATCAAGTTACTCAGGGGAAGAAGAGTCACACTCAGAGTCAACAGACTGGTCCAACGACACTGACTTCTGACAGGATTCTTGAcagttgtttcatatgaagagattgttaagtcacgataatcaataggagaaatcaa                                                                                                                                                                                                                      >  1:388930‑M1/1‑246 (MQ=255)`  
                                                                                               `tACGCCAACTATCTGATACTATTATTGCCCTTGAGCGTAATCAGCAAGGCGATATGCCTAACCTTGTCCTCGTTCGTATTCTCAAGTGCCGCTTTACTGGTGATACTGGTATCGCTGGCTACATGGAATACAACAAGGAAACCGGATGGCTTGAACCATCAAGTTACTCAGGGGAAGAAGAGTCACACTCAGAGTCAACAGACTGGTCCAACGACACTGACTTCTGACAGGATTCTTGATGACTTTCCAGACGACTACGAGAAGTTTCGCTGGAGAGTCCCATTCTAATACGACTCACTaa                                                                                                                                                                                                               <  1:429764/301‑1 (MQ=255)`  
                                                                                                `aCGCCAACTATCTGATACTATTATTGCCCTTGAGCGTAATCAGCAAGGCGATATGCCTAACCTTGTCCTCGTTCGTATTCTCAAGTGCCGCTTTACTGGTGATACTGGTATCGCTGGCTACATGGAATACAACAAGGAAACCGGATGGCTTGAACCATCAAGTTACTCAGGGGAAGAAGAGTCACACTCAGAGTCAACAGACTGGTCCAACGACACTGACTTCTGACAGGATTCTTGATGACTTTCCAGACGACTACGAGAAGTTTCGCTGgagctgtctcttatacacatctccgagccc                                                                                                                                                                                                              >  1:284830/1‑274 (MQ=255)`  
                                                                                                  `gtgtataagagacagcTATTATTGCCCTTGAGCGTAATCAGCAAGGCGATATGCCTAACCTTGTCCTCGTTCGTATTCTCAAGTGCCGCTTTACTGGTGATACTGGTATCGCTGGCTACATGGAATACAACAAGGAAACCGGATGGCTTGAACCATCAAGTTACTCAGGGGAAGAAGAGTCACACTCAGAGTCAACAGACTGGTCCAACGACACTGACTTCTGACAGGATTCTTGATGACTTTCCAGACGACTACGAGAAGTTTCGCTGGAGAGTCCCATTCTAATACGACTCACTAAAgg                                                                                                                                                                                                            <  1:363295/286‑1 (MQ=255)`  
                                                                                                  `gCCAACTATCGGATACTATTATTGCCCTTGCGCGTAATCAGCAAGGCGATATGCCTAACCTTGTCCTCGTTCGTATTCTCAAGTGCCGCTTTACTGGTGATACTGGTATCGCTGGCTACATGGAATACAACAAGGAAACCGGATGGCTTGAACCATCAAGTTACTCAGGGGAAGAAGAGTCACACTCAGAGTCAACAGACTGGTCCAACGACACTGACTTCTGACAGGATTCTTGATGACTTTCCAGACGACTACGAGAAGTTTCGCTGGAGAGTCCCATTCTAATACGACTCACTAAAgg                                                                                                                                                                                                            <  1:118203/301‑1 (MQ=255)`  
                                                                                                  `cccAACTATCTGATACTATTATTGCCCTTGATCGTAATCAGCAATGCGATATGCCTAACCTTGTCCTCGTTCGTATTCTCAAGTGCCGCTTTACTGGTGATACTGGTATCGCTTGCTACATGGAATACAACAAGGAAACCGGATGGCTTGAACCATCAAGATACTCAGGGGAAGAAGAGTCACACTCAGAGTCAACAGACTGGTCCAACGACACTGACTTCTGACAGGATTCTTGATGACTTTCCAGACGACTACGAGAAGTTTCGCTGGAGAGTCCCATTCTAATACGACTCACTAAAgg                                                                                                                                                                                                            <  1:142561/300‑1 (MQ=255)`  
                                                                                                  `aagacagtacTGATACTATTATTTCCCTTGAACGTAATCAGTAAGGCGATATGCCTAACCTTGTCCTCGTTCGTATTCTCAAGTGCCGCTTTACTGGTGATACTGGTATCGCTGGCTACATGGAATACAACAATGAAACCGGATTGCTTGAACCATCAAGTTACTCAGGAGAAGAAGAGTCACACTCAGAGTCAACAGACTGGTCCAACGACACTGACTTCTGACAGGATTCTTGATGACTTTCCAAACGACTACGAGAATTTTCGCTGGAGAGTCCCATTCTAATACGACTCACTAAAgg                                                                                                                                                                                                            <  1:23789/292‑1 (MQ=255)`  
                                                                                                      `aatgtCTGGTACTATTAGTGCCCTGTAGCGAAATCAGCAAGGTGATATGCCTAACCTTGTCCTCGTTCGTATTCTCAAGTGCAGCTTTACTGGTGATACTGGTATCGCTGGATACATGGAATACAACAAGGAAACCGGATGGCTTGAACCATCAAGTTACTCAGGGGAAGAAGAGTCACACTCAGAGTCAACAGACTGGTCCAACGACACTGACTTCTGACAGGATTCTTGATGACTTTCCAGACGACTACGAGAAGTTTCGCTGGAGAGTCCCATTCTAATACGACTCACTAAAGGAGac                                                                                                                                                                                                        <  1:371425/297‑1 (MQ=255)`  
                                                                                                      `aagaTCTGATACTATTATTGCCCTTGAGCGTAATCAGCAAGGCGATATGCCTAACATTGTCCTCGTTCGTATTCTCAAGTGCCGCTTTACTGGTGATACTGGTATCGCTGGCTACATGGAATACAACAAGGAAACCGGATGGCTTGAACCATCAAGTTACTCAGGGGAAGAAGAGTCACACTCAGAGTCAACAGACTGGTCCAACGACACTGACTTCTGACAGGATTCTTGATGACTTTCCAGACGACTACGAGAAGTTTCGCTGGAGAGTCCCATTCTAATACGACTCACTAAAGGAGac                                                                                                                                                                                                        <  1:200295/298‑1 (MQ=255)`  
                                                                                                       `cTATCTGATACTATTATTGCCCTTGAGCGTCATCAGCAAGGCGATATGCCTAACCTTGTCCTCGTTCGTATTCTCCAGTGCCGCTTTACTGGTGATACTGGTATCGCTGGCGACATGTAATACAACACGTAAACCGGATGGCTTGAACCATCAAGTTACTCAGGGGAAGAAGAGTCACACTCAGAGTCAACAGACTGGTCCAACGACACTGACTTCTGACAGGATTCTTGAcagttgcttcatatgaagagattgttaagtcacgataacccataggagaaatcaatatgatcgtttctga                                                                                                                                                                                                       >  1:375283‑M1/1‑231 (MQ=255)`  
                                                                                                       `cTATCTGATACTATTATTGCCCTTGAGCGTAATCAGCAAGGCGATATGCCTAACCTTGTCCTCGTTCGTATTCTCAAGTGCCGCTTTACTGGTGATACTGGTATCGCTGGCTACATGGAATACAACAAGGAAACCGGATGGCTTGAACCATCAAGTTACTCAGGGGAAGAAGAGTCACACTCAGAGTCAACAGACTGGTCCAACGACACTGACTTCTGACAGGATTCTTGAcagttgtttcatatgaagagattgttaagtcacgataatcaatcggagaaatcaatatgatcgtttctga                                                                                                                                                                                                       >  1:138104‑M1/1‑231 (MQ=255)`  
                                                                                                        `aaTCTGATACTATTATTGCCCTTGAGCGTAATCAGCAAGGCGATATGCCTAACCTTGTCCTCGTTCGTATTCGCAAGTGCCGCTTTACTGGTGATACTGGTATCGCTGGCTACATGGAATACAACAAGGAAACCGGATGGCTTGAACCATCAAGTTACTCAGGGGAAGAAGAGTCACACTCAGAGTCAACAGACTGGTCCAACGACACTGACTTCTGACAGGATTCTTGATGACTTTCCAGACGACTACGAGAAGTTTCGCTGGAGAGTCCCATTCTAATACGACTCACTAAAGGAGacac                                                                                                                                                                                                      <  1:426314/300‑1 (MQ=255)`  
                                                                                                         `ttcggatatgattattGCCCTTGAGCGGAATCAGCAAGGCGATATGCCTAACCTTGTCCTCGTTCGTATTCTCAAGTGCCGCGTTACTGGTGATACTGGTATCGCTGGCTACATGGAATACAACAAGGAAACCGGATGGCTTGAACCATCAAGTTACTCAGGGGAAGAAGAGTCACACTCAGAGTCAACAGACTGGTCCAACGACACTGACTTCTGACAGGATTCTTGATGACTTTCCAGACGACTACGAGAAGTTTCGCTGGAGAGTCCCATTCTAATACGACTCACTAAAGGAGacaca                                                                                                                                                                                                     <  1:91568/291‑1 (MQ=255)`  
                                                                                                         `aTCTGATACTATTATTGCCCTTGAGCGTAATCAGCAAGGCGATATGCCTAACCTTGTCCTCGTTCGTATTCTCAAGTGCCGCTTTACTGGTGATACTGGTATCGCTGGCTACATGGAATACAACAAGGAAACCGGATGGCTTGAACCATCAAGTTACTCAGGGGAAGAAGAGTCACACTCAGAGTCAACAGACTGGTCCAACGACACTTACTTCTGACAGGATTCTTGATGACTTTCAAGACGACTACGAGAAGTTTCGCTGGAGAGTCCCATTCTAATACGACTCACTAAAGGAGacaca                                                                                                                                                                                                     >  1:226754/1‑301 (MQ=255)`  
                                                                                                         `aTCTGATACTATTATTGCCCTTGAGCGTAATCAGCAAGGCGATATGCCTAACCTTGTCCTCGTTCGTATTCTCAAGTGCCGCTTTACTGGTGATACTGGTATCGCTGGCTACATGGAATACAACAAGGAAACCGGATGGCTTGAACCATCAAGTTACTCAGGGGAAGAAGAGTCACACTCAGAGTCAACAGACTGGTCCAACGACACTGACTTCTGACAGGATTCTTGATGACTTTCCAGACGACTACGAGAAGTTTCGCTGGAGAGTCCCATTCTAATACGACTCACTAAAGGAGacaca                                                                                                                                                                                                     >  1:393501/1‑301 (MQ=255)`  
                                                                                                                `aCTATTATTGCCATTGAGCGTAATCAGCAAGGCGATATGCCTAACCTTGTCATCGTTAGTATTATCAAGTGCCGCTGTACTGGTGATACTGGTATCGCTTGCTACATGGAATACAAAAAGGAACCCGTATGGCTTGAACCATCAAGTTACTCAGGGGAAGAAGAGTCACACTCAGAGTCAACATACTGGTCCAACGACACTTACTTCTGACAGGATTCTTGAcagttgtttcatatgaagagattgttaagtcacgataatcaataggagaaatcaatatgatcgtttctgacatcgaagc                                                                                                                                                                                              <  1:306621‑M1/301‑80 (MQ=255)`  
                                                                                                                 `cTATTATTGCCCTTGAGCGTAATCAGCAAGGCGATATGCCTAACCTTGTCCTCGTTCGTATTCTCAAGTGCCGCTTTACTGGTGATACTGGTATCGCTGGCTACATGGAATACAACAAGGAAACCGGATGGCTTGAACCATCAAGTTAATCAGGGGAAGAAGAGTCACACTCAGAGTCAACAGACTGGTACTACGACACTGACTTCTGACAGGATTCTTGATGACTTTCCAGACGACTACGAGAAGTTTCGCTGGAGAGTCCCATTCTAATACGACTCACTAAAGGAGAAACACCATGTTc                                                                                                                                                                                             >  1:106789/1‑301 (MQ=255)`  
                                                                                                                   `attattGCCCTTGAGCGTAATCAGCAAGGCGATATGCCTAACCTTGTCCTCGTTCGTATTCTCAAGTGCCGCTTTACTGGTGATCCTGGTATCGCTGGCTACATGGAATACAACAAGGAAACCGGATGGCTTGAACCATCAAGTTACTCAGGGGAAGAAGAGTCACACTCAGAGTCAACAGACTGGTCCAACGACACTGACTTCTGACAGGATTCTTGATGACTTTCCAGACGACTACGACAAGTTTCGCTGGAGAGTCCCATTCTAATACGACTCACTCAAGGAGACACACCATGTTCaa                                                                                                                                                                                           >  1:163312/1‑301 (MQ=255)`  
                                                                                                                   `attattGCCCTTGAGCGTAATCAGCAAGGCGATATGCCTAACCTTGTCCTCGTTCGTATTCTCAAGTGCCGCTTTACTGGTGATACTGGTATCGCTGGCTACATGGAATACAACAAGGAAACCGGATGGCTTGAACCATCAAGTTACTCAGGGGAAGAAGAGTCACACTCAGAGTCAACAGACTGGTCCAACGACACTGACTTCTGACAGGATTCTTGATGACTTTCCAGACGACTACGAGAAGTTTCGCTGGAGAGTCCCATTCTAATACGACTCACTAAAGGAGACACACCATGTTCaa                                                                                                                                                                                           >  1:463660/1‑301 (MQ=255)`  
                                                                                                                     `tattGCCCTTGAGCGTAATCAGCAAGGCGATATGCCTAACCTTGTCCTCGTTCGTATTCTCAAGTGCCGCTTTACTGGTGATACTGGTATCGCTGGCTACATGGAATACAACAAGGAAACCGGATGGCTTGAACCATCAAGTTACTCAGGGGAAGAAGAGTCACACTCAGAGTCAACAGACTGGTCCAACGACACTGACTTCTGACAGGATTCTTGATGACTTTCCAGACGACTACGAGAAGTTTCGCTGGAGAGTCCCATTCTAATACGACTCACTAAAGGAGACACACCATGTTCAAAc                                                                                                                                                                                         <  1:348452/301‑1 (MQ=255)`  
                                                                                                                               `gAGCGTAATCAGCAAGGGGAGATGCCTAACCTTGTCCTCGTTCGTATTCTCAAGTGCCGCTTTACTGGTGATACTGGTATCGCTGGCTACATGGAATACAACAAGGAAACCGGATGGCTTGAACCATCAAGTTACTCAGGGGAAGAAGAGTCACACTCAGAGTCAACAGACTGGTCCAACGACACTGACTTCTTACAGGATTCTTGATGACTTTCCAGACGACTACGAGAAGTTTCGCTGGAGAGTCCCATTCTAATACGACTCACTAAAGGAGACACACCATGTTCAAACTGATTaagaa                                                                                                                                                                               <  1:420897/301‑1 (MQ=255)`  
                                                                                                                                `ggcGTAATCAGCAAGGCGATATGCCTAACCTTGTCCTCGTTCGTATTCTCAAGTGCCGCTTTACTGGTGATACTGGTATCGCTGGCTACATGGAATACAACAAGGAAACCGGATGGCTTGAACCATCAAGTTACTCAGGGGAAGAAGAGTCACACTCAGAGTCAACAGACTGGTCCAACGACACTGACTTCTGACAGGATTCTTGAcagttgtttcatatgaagagattgttaagtcacgataatcaataggagaaatcaatatgatcgtttctgacatcgaagctaacgccctcttagag                                                                                                                                                                              <  1:8548‑M1/300‑96 (MQ=255)`  
                                                                                                                                `aGCGTAATCAGCAAGGCGATATGCCTAACCTTGTCCTCGTTCGTATTCTCAAGTGCCGCTTTACTGGTGATACTGGTATCGCTGGCTACATGGAATACAACAAGGAAACCGGATGGCTTGAACCATCAAGTTACTCAGGGGAAGAAGAGTCACACTCAGAGTCAACAGACTGGTCCAACGACAATGACGTCTGACAGGATTCTTGATGACTTTCCAGACGACTACTAGAAGTTTCGCTGGAGAGTCCCATTCTAATACGACTCACTAAAGGAGACACACCATGTTCAAACTGATTaagaaa                                                                                                                                                                              >  1:417142/1‑300 (MQ=255)`  
                                                                                                                                `aGCGTAATCAGCAAGGCGATATGCCTAACCGTGTCCTCGTTCGTATTCTCAAGTGCCGCTTTAATGGTGATAATGGTATCGCTGGCTACATGGAATACAACAAGGAAACCGGATGGCTTGAACCATCAAGTTACTCAGGGGAAGAAGAGTCACACTCAGAGTCAACAGACTGGTACAACGACACTGACTTCTGACAGGATTCTTGAcagttgtttcatatgaagagattgttaagtcacgataatcaataggagaaatcaatatgatcgtttctgacatcgaagctaacgccctcttagag                                                                                                                                                                              <  1:241933‑M1/301‑96 (MQ=255)`  
                                                                                                                                  `cGTAATCAGCAAGGCGCTATGCCTAACCTTGTCCTCGTTCGTATTCTCAAGTGCCGATTTACTGGTGATAATGGTATCGCTGGCTACATGGAATACAACAAGGAACCCGGATGGCTTGAACCATCAAGTTACTCAGGGTAAGAAGAGTCACACTCAGAGTCAACAGACTGGTCCAACGACACTGACTTCTGACAGGATTCTTGATGACTTTCCAGACGACTACGAGAAGTTTCGCTGGAGAGTCCCATTCTAATACGACTCACTAAAGGAGACACACAATGTTCaaaactgtcgcttataa                                                                                                                                                                            >  1:55633/1‑287 (MQ=255)`  
                                                                                                                                   `gTAATCAGCAAGGCGATATGCCTAACCTTGTCCTCGTTCGTATTCTCAAGTGCCGCTTTACTGGTGATACTGGTATCGCTGGCTACATGGAATACAACAAGGAAACCGGATGGCTTGAACCATCAAGTTACTCAGGGGAAGAAGAGTCACACTCAGAGTCAACAGACTGGTCCAACGACACTGACTTCTGACAGGATTCTTGATGACTTTCCAGACGTCTATGAGAAGTTTCGCTGGAGAGTCCAATTCTAATACGACTCATTAAAGGAGAAACACCATTTTCAACATGTTTAAGAAGTTa                                                                                                                                                                           >  1:317192/1‑301 (MQ=255)`  
                                                                                                                                   `gTAATCAGCAAGGCGATATGCCTAACCTTGTCCTCGTTCGTATTCTCAAGTGCCGCTTTACTGGTGATACTGGTATCGCTGGCTACATGGAATACAACAAGGAAACCGGATGGCTTGAACCATCAAGTTACTCAGGGGAAGAAGAGTCACACTCAGAGTCAACAGACTGGTCCAACGACACTGACTTCTGACAGGATTCTTGATGACTTTCCAGACGACTACGAGAAGTTTAGCTGGAGAGTCCCATTCTAATACGACTCACTAAAGGAGACACACCATGTTCAAACTGATTAAGAAGTTa                                                                                                                                                                           >  1:359015/1‑301 (MQ=255)`  
                                                                                                                                     `aaTCAGGAAGGATAGATGCCTAACCTTGTCCTCGTTCGTATGTTTAAGAGCCGCGTTACTGGTGAGACTGGTATCGCTGGCTACATGGAATACAACAAGGAAACCTGATGGCTTGAACCATCAAGTTACTCAGGGGAAGAAGAGTCACACTCAGAGTCAACAGACTGGTCCAACGACACTGACTTCTGACAGGATTCTTGATTACTTTCCAGACGACTACGAGAAGTTTCGCTGGAGAGTCCCATTCTAATACGACTCACTAAAGGAGACACACCATGTTCAAACTGATTAAGAAGTTAgg                                                                                                                                                                         <  1:497550/301‑1 (MQ=255)`  
                                                                                                                                      `aTCAGCAAGGCGATATGCCGAACCGTGTCCTCGTTCGTATTCTCAAGTGCCGCTTTACTGGTGATACTGGTATCGCTGGCTACATGGAATACAACAAGGAAACCGGATGGCTTGAACCATCAAGTTACTCAGGGTAAGAAGAGTCACACTCAGAGTCAACAGACTGGTCCAACGACACTGACTTCTGACAGGATTCTTGATGACTTTCCAGACGACTACGAGAAGTTTCGCTGGAGAGTCCCATTCTAATACGACTCACTAACGGAGACACACCATTTTCAAACTGATTAAGAAGTTAGGc                                                                                                                                                                        <  1:311105/301‑1 (MQ=255)`  
                                                                                                                                                  `agatgtgtataagagacagcgttcgtATTCTCAAGTGCCGCTTTACTGGTGATACTGGTATCGCTGGCTACATGGAATACAACAAGGAAACCGGATGGCTTGAACCATCAAGTTACTCAGGGGAAGAAGAGTCACACTCAGAGTCAACAGACTGGTCCAACGACACTGACTTCTGACAGGATTCTTGATGACTTTCCAGACGACTACGAGAAGTTTCGCTGGAGAGTCCCATTCTAATACGACTCACTAAAGGAGACACACAATGTTCAAACTGATTAAGAAGTTAGGCCAACTGCTGGtt                                                                                                                                                            <  1:332959/282‑1 (MQ=255)`  
                                                                                                                                                     `tGCCTAACCTTGTCCTCGTTCGTATTCTCAAGTGCCGCTTTACTGGTGATACTGGTATCGCTGGCTACATGGAATACAACAAGGAAACCGGATGGCTTGAACCATCAAGTTACTCCGGGGAAGAAGAGTCACACTCAGAGTCAACAGACTGGTCCAACGACACTGACTTCTGACAGGATTCTTGATGACTTTCCAGACGACTACGAGAAGTTTCGCTGGAGAGTCCCATTCTAATACGACTCACTAAAGGAGACACACCATGTTCAAACTGATTAAGAAGTTAGGCCAACTGCTGGTTCGt                                                                                                                                                         >  1:482416/1‑301 (MQ=255)`  
                                                                                                                                                      `gCCTAACCTTGTCCTCGTTCGTATTCTCAAGTGCCGCTTTACTGGTGATACTGGTATCGCTGGCTACATGGAATACAACAAGGAAACCGGATGGCTTGAACCATCAAGTTACTCAGGGGAAGACGAGTCACACTCAGAGTCAACAGACTGGTCCAACGACACTGACTTCTGACAGGATTCTTGATGACTTTCCAGACGACTACGAGAAGTTTCGCTGGAGAGTCCCATTCTAATACGACTCACTAAAGTAGACACACCATGTTCACACTGATTAAGAAGTTAGGCCAACTGCTGGTTCGtt                                                                                                                                                        >  1:65103/1‑300 (MQ=255)`  
                                                                                                                                                      `gCCTAACCTTGTCCTCGTTCGTATTCTCAAGTGCCGCTTTACTGGTGATACTGGTATCGCTGGCTACATGGAATACAACAAGGAAACCGGATGGCTTGAACCATCAAGTTACTCAGGGGAAGAAGAGTCACACTCAGAGTCAACTTACTGGTCCAACGACACTTACTTCTGACAGGATTCTTGAcagttgtttcatatgaagagattgttaagtcacgataatcaataggagaaatcaatatgatcgtttctgacatcgaagctaacgccctcttagagagcgtcactaagttcctgtctt                                                                                                                                                        >  1:3793‑M1/1‑184 (MQ=255)`  
                                                                                                                                                      `gCCTAACCTTGTCCTCGTTCGTATTCTCAAGTGCCGCTTTACTGGTGATACTGGTATCGCTGGCTACATGGAATACAACAAGGAAACCGGATGGCTTGAACCATCAAGTTACTCAGGGGAAGAAGAGTCACACTCAGAGTCAACAGACTGGTCCAACGACACTGACTTCTGACAGGATTTTTGATGACTTTCCAGACGACTACGAGCAGTTTCGCTGTAGAGTCCCATTCTAATACGACTCACTAAAGGAGACACACCATGTTCAAACTGATTAAGAAATTAGTCCAACTGCTGGTTCGTa                                                                                                                                                        >  1:412082/1‑301 (MQ=255)`  
                                                                                                                                                       `gcTAAACTTGTCATCGTTCGTAATATCAAGTGCAGCTGTAAAGGGGATACTGGGATCGCTGGCTACATGGAAGACAACAAGGAAATCGGATGGCTTGAAACATCAAGTTACGAAGGGGAAGAAGAGTCACAATAAGAGTCAACAGACTGGTCCAACGACAATGACTTCTGACAGGATTCTTGAAGACTTTCCAGACGACTACGAGAAGTTTCGCTGGAGAGGAACAGTATAATACGACTCACTAAAGGAGAAACACCATGTTCAAACTGATTAAGAAGTTAGGCCAACTGCTGGTTCGTAt                                                                                                                                                       <  1:253741/300‑1 (MQ=255)`  
                                                                                                                                                                   `cTCGTTCGAATTCTCAAGTGCCGCTTTACTGGTGATACTGGTATCGCTGGCTACATGGAATACAACAAGGAAACCGGATGGCTTGAACCATCAAGTTACTCAGGGGAAGAAGAGTAACACTCAGAGTCAACAGACTGGTCCAACGACACTGACTTCTGACAGGATTCTTGATGACTTTCCAGACGACTACGAGAAGTTTCGCTGGAGAGTCCCATTCTAATACGACTCACTAAAGGAGACACACCATGTTCAAACTGATTAAGAAGTTAGGCCAACTGCTGGTTCGTATGTACAACGTGGa                                                                                                                                           <  1:18676/301‑1 (MQ=255)`  
                                                                                                                                                                    `tcgttcgtATTCTCAAGTGCCGCTTTACTGGTGATACTGGTATCGCTGGCTACATGGAATACAACAAGGAAACCGGATGGCTTGAACCATCAAGTTACTCAGGGGAAGAAGAGTCACACTCAGAGTCAACAGACTGGTCCAACGACACTGACTTCTGACAGGATTCTTGAcagttgtttcatatgaagagattgttaagtcacgataatcaataggagaaatcaatatgatcgtttctgacatcgaagctaacgccctcttagagagcgtcactaagttccactgcggggttatctacgac                                                                                                                                          <  1:133064‑M1/301‑132 (MQ=255)`  
                                                                                                                                                                            `gtataagagacaggcattACTGGTGAGACTGGTATCTCTGGCTACATGGAATACAACAAGGAAACCGGATGGCTTGAACCATCAAGTTACTCAGGGGAAGAAGAGTCACACTCAGAGTCAACAGACTGGTCCAACGACACTGACTTCTGACAGGATTCTTGATGACTTTCCAGACGACTACGAGAAGTTTCGCTGGAGAGTCCCATTCTAATACGACTCGCTAAAGGAGACACACCATGTTCAAACTGATTAAGAAGTTAGGCCAACTGCTGGTTCGTATGTACAACGTGGAAGCCAAGCg                                                                                                                                  <  1:174124/285‑1 (MQ=255)`  
                                                                                                                                                                            `aTTCTCAAGTGCCGCTTTACTGGTGATACTGGTATCGCTGGCTACATGGAATACAACAAGGAAACCGGATGGCTTGAACCATCAAGTTACTCAGGGGAAGAAGAGTCACACTCAGAGTCAACAGACTGGTCCAACGACACTGACTTCTGACAGGATTCTTGATTACTTTCCAGACGACTACGAGAAGTTTCGCTGGAGAGTCCCATTCTAATACGACTCACTAAAGGAGACACACCATGTTCAAACTGATTAAGAAGTTAGGCCCACTGCTGGTTCGTATGTACAACGTGGAAGCCAAGCg                                                                                                                                  >  1:263882/1‑301 (MQ=255)`  
                                                                                                                                                                            `aTTCTCAAGTGCCGATTTACTGGTGATACTGGTATCGCTGGCTACATGGAATACAACAAGGAAACCGGATGGCTTGAACCATCAAGTTACTCAGGGGAAGAAGAGTCACACTCAGAGTCAACAGACTGGTCCAACGACACTGACTTCTGACAGGATTCTTGAcagttgtttcatatgaagagattgttaagtcacgataatcaataggagaaataaatatgatcgtttctgacatcgaagctaacgccctcttagagagcgtcactaagttccactgcggggttatctacgacctgtctct                                                                                                                                  >  1:95196‑M1/1‑162 (MQ=255)`  
                                                                                                                                                                                   `aGTGCCGCTTTACTGGTGATACTGGTATCGCTGGCTACATGGAATACAACAAGGAAACCGGATTGCTTGAACCATCAAGTTACTCAGGGGAAGAAGAGTCACACTCAGAGTCAACAGACTGGTCCAACGACACTGACTTCTGACAGGATTCTTGAcagttgttccatatgaagagattgttaagtcacgataatcaataggagaaatcaatatgatcgtttctgacatcgaagctaacgccctcttagagagcgtcactaagttccactgcggggttatctacgactactccaccgctgag                                                                                                                           <  1:234405‑M1/301‑147 (MQ=255)`  
                                                                                                                                                                                      `cccgCGTTACTGGTGAGACGGGTATGGCTGGCTAAATGGAATAAAACAAGGAAACCGGATGGCTTGAAACATCAAGTTACTCAGGGGAAGAAGAGTCACACTCAGAGTAAACAGACTGGTCCAACGACACTAACTTCTGACAGGATTCTTGAcagttgtttcatatgaagagattgttaagtcacgataatcaataggcgaaatcaatatgatcgtttctgacatcgaagctaacgccctcttagagagcgtcactaagttccactgcggggttatctacgactactccaccgctgagtac                                                                                                                        <  1:321993‑M1/300‑150 (MQ=255)`  
                                                                                                                                                                                        `agcgatACCGGTGATACTGGTATCGCTGGCTACATGGAATACAACAAGGAAACCGGATGGCTTGAACCATCAAGTTACTCAGGGGAAGAAGAGTCACACTCAGAGTCAACAGACTGGTCCAACGACACTGACTTCTGACAGGATTCTTGATGACTTTCCAGACGACTACGAGAAGTTTCGCTGGAGAGTCCCATTCTAATACGACTCACTAAAGGAGACACACCATGTTCAAACTGATTAAGAAGTTAGGCCAACTGCTGGTTCGTATGTACAACGTGGAAGCCAAGCGACTGAACGATGa                                                                                                                      <  1:10070/296‑1 (MQ=255)`  
                                                                                                                                                                                        `aagagacagggTGATACTGGTATCGCTGGCTACATGGAATACAACAAGGAAACCGGATGGCTTGAACCATCAAGTTACTCAGGGGAAGAAGAGTCACACTCAGAGTCAACAGACTGGTCCAACGACACTGACTTCTGACAGGATTCTTGATGACTTTCCAGACGACTACGAGAAGTTTCGCTGGAGAGTCCCATTCTAATACGACTCACTAAAGGAGACACACCATGTTCAAACTGATTAAGAAGTTAGGCCAACTGCTGGTTCATATGTACAACGTGGAAGCCAAGCGACTGAACGATGa                                                                                                                      <  1:448295/292‑1 (MQ=255)`  
                                                                                                                                                                                          `cTTTACTGGTGATACTGGTATCTCTGGCAACATGGAATACAACAAGGAAACCGGATGGCTTGAACCATCAAGTTACTCAGGGGAAGAAGAGTCACACTCAGAGTCAACAGACTGGTCCAACGACACTGACTTCTGACAGGATTCTTGATGACTTTCCAGACGACTACGAGAAGTTTCGCTGGAGAGTCCCATTCTAATACGACTCCCTAAAGGAGACACACCATGTTCAAACTGATTAAGAAATTAGGCCAACTGCTGGTTCGTATGTACAACGTGGAAGCCAAGCGACTGAACGATGAgg                                                                                                                    >  1:73565/1‑301 (MQ=255)`  
                                                                                                                                                                                          `cTTTACTGGTGATACTGGTATCGCTGGCCACATGGAATACAACAAGGAAACCGGATGGCTTGAACCATCAAGTTACTCAGGGGAAGAAGAGTCACACTCAGAGTCAACAGACTGGTCCAACGACACTGACTTCTGAAAGGACTCTTGAcagttgtttcatatgaagagattgttaagtcacgttaatcaataggagaaatcaatctgatcgtttctgacatcgacgataacgccctattagagagcgtcactaagttccactgcgggtttttctccgacctggccctttaccacatacacc                                                                                                                    >  1:80851‑M1/1‑148 (MQ=255)`  
                                                                                                                                                                                           `tttACTGGTGATACTGGTATCGCTGGCTACATGGAATACAACAAGGAAACCGGATGGCTTGAACCATCAAGTTACTCAGGGGAAGAAGAGTCACACTCAGAGTCAACAGACTGGTCCAACGACACTGACTTCTGACAGGATTCTTGAcagttgtttcatatgaagagattgttaagtcacgataatcaataggagaaatcaatatgatcgtttctgacatcgaagctaacgccctcttagagagcgtcactaagttccactgcggggttatctacgactactccaccgctgagtacgtaag                                                                                                                   <  1:52050‑M1/301‑155 (MQ=255)`  
                                                                                                                                                                                                 `tatgatactttcatatatGGCTACATGTAAGTAAAAAAAGAAACTGGATGTCTTGAACCAACAAATTACTCAGGGGAAGAAGAGACACACTCAGAGTCAACAGACTGGTCCAACGACACTGACTTCTGACAAGATTCTGTATAACTTTCCAGACTACTACGAGAAGTTTCGCTGGAGAAGCCCATTCTTATACGAGTAACTAAAGGAGACACACCATGTTCAAACTGATTAAGAAGTTGGGCCAACTGCTTGTTCGTATGTACAACGTGGAAGCCAAGCGACTGAACGATGAGGCGCGTaa                                                                                                             <  1:247449/284‑1 (MQ=255)`  
                                                                                                                                                                                                 `aacgtacagggtatcgcgggCTAAATGGACTACAACAAGGAAACCGGATGGCTTGAACCATCAAGTTACTCAGGGGAAGAAGAGTCACACTCAGAGTCAACAGACTGGTCCAACGACACTTACTTCTGACAGGTTTCTTGAcagttgtttcatatgaagagattgttaagtcacgataatcaataggagaaatcaatatgatcgtttctgacatcgaagctaacgccctcttagagagcgtcactaagttccactgcggggttatctacgactactccaccgctgagtacgtaagctaccg                                                                                                             <  1:337958‑M1/292‑161 (MQ=255)`  
                                                                                                                                                                                                   `tGATACTGGTATCGCTGGCTACATGGAATACAACAAGGAAACCGGATGGCTTGAACCATCAAGTTAATCAGGGGAAGAAGAGTCACACTCAGAGTCAACAGACTGGTCCAACGACACTGACTTCTGACAGGATTCTTGATGACTTTCCAGACGACTACGAGAAGTTTCGCTGGAGAGTCCCATTCTAATACGACTCACTAAAGGAGACACACCATGTTCAAACTGATTAAGAAGTTAGGCCAACTGCTGGTTCGTATGTACAACGTGGAAGCCAAGCGACTGAACGATGAGGCTCGTAAag                                                                                                           <  1:403984/301‑1 (MQ=255)`  
                                                                                                                                                                                                   `ggaatcTGGGATTGCTGGCTACAAGTAATACAAAAAAGAAACCGGATGGCTTGAACCATCAAGTTACTACGGGGAAGAAGAGTAACACTCAGAGTCAACAGACTGGGCCAACGACACTAACTTCTGACAGGATTCTTGATGACGTTCCAGACGACTACGAGAAGTTTCGCTGGAGAGTCCCATTCTAATACGACTAACTAAAGGAGACAAACCATGTTCAAACTGATTAAGAAGTTAGGCCAACTGCTGGTTCGTATGTACAAAGTGGAAGCCAAGCGACTGAACGATGAGGCTCGTAAag                                                                                                           <  1:300739/296‑1 (MQ=255)`  
                                                                                                                                                                                                   `ataagagaaagtCGCTGGCAAAAAGGAATACAACAAGGAAACCGGATGGCTTGAACCATCAAGTTACTCAGGGGAAGAAGAGTCACACTCAGAGTCAATAGACGGGTCCAACGACACTGACTTCTGACAGGATTCTTGATGACTTTCCAGACGACTACGAGAAGTTTCGCTGGAGAGTCCCATTCTAATACGACTCACTAAAGGAGACACACCATGTTCAAACTGATTAAGAAGTTAGGCCAACTGCTGGTTCGTATGTACAACGTGGAAGCCAAGCGACTGAACGATGAGGCTCGTAAag                                                                                                           <  1:61391/290‑1 (MQ=255)`  
                                                                                                                                                                                                    `gATACTGGTATCGCTGGCTACATGGAATACAACAAGGAAACCGGATGGCTTGAACCATCAAGTTACTCAGGGGAAGAAGAGTCACACTCAGAGTCAACAGACTGGTCCAACGACACTGACTTCTGACAGGATTCTTGAcagttgtttcatatgaagagattgttaagtcacgataatcaataggagaaatcaatatgatcgtttctgacatcgaagctaacgccctcttagagagcgtcactaagttccactgcggggttatctacgactactccaccgctgagtacgtaactgtctctta                                                                                                          >  1:443962‑M1/1‑138 (MQ=255)`  
                                                                                                                                                                                                            `aaTCGCCGGCTAAATAAAAAACAAAAAGGAAACCGGATGGCTACAACCATCAAGTTACTCAAGGGAAGAAGAGTCACACTCATAGTCAACAGAATGGTCCAACGACCCTGACTTCTGACAGGATTCGTGATGACTTTCCAGACGACTACGAGAAGTTTCGCTGGCGATTCCCATTCTAATACGACTCACTAAAGGAGACACACCATGTTCAAACTGATTAAGAAGTTAGGCCAACTGCTGGTTCGTATGTACAACGTGGAAGCCAAGCTACTGAACGATGAGGCTCGTAAAGAGGCcacac                                                                                                  <  1:110719/300‑1 (MQ=255)`  
                                                                                                                                                                                                                         `aTGGAATACAACAAGGAAACCGGATGGCTTGAACCATCAAGTTACTCAGGGGAAGAAGAGTCACACTCAGAGTCAACAGACTGGTCCAACGACACTGACTTCTGACAGGATTCTTGATGACTTTCCAGACGACTACGAGAAGTTTCGCTGGAGAGTCCCATTCTAATACGACTCACTAAAGGAGACACACCATGTTCAAACTGATTAAGAAGTTAGGCCAACTGCTGGTTCGTATGTACAACGTGGAAGCCAAGCGACTGAACGATGAGGCTCGTAAAGAGGCCACACAGTCACgcgcctg                                                                                     >  1:155714/1‑298 (MQ=255)`  
                                                                                                                                                                                                                            `gAATACAACAAGGAAACCGGATGGCTTGAACCATCAAGTTACTCAGGGGAAGAAGAGTCACACTCAGAGTCAACAGACTGGTCCAACGACACTGACTTCTGACAGGATTCTTGATGACTTTCCAGACGACTACGAGAAGTTTCGCTGGAGAGTCCCATTCTAATACGACTCACTAAAGGAGACACACCATGTTCAAACTGATTAAGAAGTTAGGCCAACTGCTGGTTCGTATGTACAACGTGGAAGCCAAGCGACTGAACGATGAGGCTCGTAAAGAGGCCACACAGTCACGCGCTCTGGc                                                                                  >  1:460486/1‑301 (MQ=255)`  
                                                                                                                                                                                                                            `gAATACAACAAGGAAACCGGATGGCTTGAACCATCAAGTTACTCAGGGGAAGAAGAGTCACACTCAGAGTCAACAGACTGGTCCAACGACACTGACTTCTGACAGGATTCATGATGACTTTCCAGACGACTACGATAAGTTTCGCTGGTGAGTCCCATTCTAATACGACTCACTAAAGGAGACACACCATGTTCAAACTGATTAAGAAGTTAGGCCAACTGCTGGTTCGTATGTACAACGTGGAAGCCAAGCGACTGAACGATGAGGCTCGTAAAGAGGCCACACAGTCACGCGCTCTGGc                                                                                  >  1:473670/1‑301 (MQ=255)`  
                                                                                                                                                                                                                              `ttcccctgagtaacttgatgggCTTGAACCATCAAGTTACTCAGGGGAAGAAGAGTCACACTCAGAGTCAACAGACTGGTCCAACGACACTGACTTCTGACAGGATTCTTGATGACTTTCCAGACGACTACGAGAAGTTTCGATGGAGAGTCCCATTCTAATACGACTCACTAAAGGAGACACACCATGTTAAAACTGATTAAGAAGTTAGGACAACTGCTGGTTCGTATGTACAACGTGGAAGCCAAGCGACTGAACGATGAGGCTCGTAAAGAGGCCACACAGTCACGCGCTCTGGCGa                                                                                <  1:221122/281‑1 (MQ=255)`  
                                                                                                                                                                                                                                    `caaGGAAACCGGATGGCTTGAACCATCAAGTTACTCAGGGGAAGAAGAGTCACACTCAGAGTCAACAGACTGGTCCAACGACACTGACTTCTGACAGGATTCTTGATGACTTTCCAGACGACTACGAGAAGTTTCGCTGGAGAGTCCCATTCTAATACGACTCACTAAAGGAGACACACCATGTTCAAACTGATTAAGAAGTTAGGCCAACTGCTGGTTCGTATGTACAACGTGGAAGCCAAGCGACTGAACGATGAGGCTCGTAAAGAGGCCACACAGTCAcgactgtctcttatccaca                                                                          >  1:195970/1‑284 (MQ=255)`  
                                                                                                                                                                                                                                      `aGGAAACCGGATGGCTTGAACCATCAAGTTACTCAGGGGAAGAAGAGTCACACTCAGAGTCAACAGACTGGTCCAACTACACTGACTTCTGACAGGATTCTTGATGACTTTCCAGACGACTACGAGAAGTTTCGCTGGAGAGTCCCATTCTAATACGACTCACTAAAGGAGACACACCATGTTCAAACTGATTAAGAAGTTAGGCCAACTGCTGGTTCGTATGTACAACGTGGAAGCCAAGCGACTGAACGATGAGGCTCGTAAAGAGGCCACACAGTCACGCGCTCTGGCGATTCGCTcc                                                                        <  1:273021/301‑1 (MQ=255)`  
                                                                                                                                                                                                                                                       `ttataagagacagaCTCAGGGGAAGAAGAGTCACACTCAGAGTCAACAGACTGGTCCAACGACACTGACTTCTGACAGGATTCTTGATGACTTTCCAGACGACTACGAGAAGTTTCGCTGGAGAGTCCCATTCTAATACGACTCACTAAAGGAGACACACCATGTTCAAACTGATTAAGAAGTTAGGCCAACTGCTGGTTCGTATGTACAACGTGGAAGCCAAGCGACTGAACGATGAGGCTCGTAAAGAGGCCACACAGTCACGCGCTCTGGCGATTCGCTCCAACGAACTGGCTGACAg                                                       <  1:407642/288‑1 (MQ=255)`  
                                                                                                                                                                                                                                                          `ccATCAAGTTACTCAGGGGAAGAAGAGTCACACTCAGAGTCAACAGACTGGTCCAACGACACTGACTTCTGACAGGATTCTTGATGACTTTCCAGACGACTACGAGAAGTTTCGCTGGAGAGTCCCATTCTAATACGACTCACTAAAGGAGACACACCATGTTCAAACTGATTAAGAAGTTAGGCCAACTGCTGGTTCGTATGTACAACGTGGAAGCCAAGCGACTGAACGATGAGGCTCGTAAAGAGGCACCACAGTCACGCGCTCTGGCGATTCGCTCAAACGAACTGGCTGACAGTGc                                                    >  1:342495/1‑301 (MQ=255)`  
                                                                                                                                                                                                                                                             `acAAGTTACTCAGGGGAAGAAGAGTCACACTCAGAGTCAACAGACTGGTCCAACGACACTGACTTCTGACAGGATTCTTGATGACTTTCCAGACGACTACGAGAAGTTTCGCTGGAGAGTCCCATTCTAATACGACTCACTAAAGGATACACACCATGTTCAAACTGATTAAGAAGTTAGGCCAACTGCTGGTTCGTATGTACAACGTGGAAGCCAAGCGACTGAACGATGAGGCTCGTAAAGAGGCCACACAGTCACGCGCTCTGGCGATTCGCTCCAACGAACTGGCTGACAGTGCATc                                                 <  1:217403/300‑1 (MQ=255)`  
                                                                                                                                                                                                                                                               `aaGTTACTCAGGGGAAGAAGAGTCACACTCAGAGTCAACAGACTGGTCCAACGACACTGACTTCTGACAGGATTCTTGATGACTTTCCAGACGACTACGATAAGTTTCGCTGGAGAGTCCCATTCTAATACGACTCACTAAAGGAGACACACCATGTTCAAACTGATTAAGAAGTTAGGCCAACTGCTGGTTCGTATGTACAACGTGGAAGCCAAGCGACTGAACGATGAGGCTCGTAAAGAGGCCACACAGTCACGCGCCCTGGCGATTCGCCCCAACGAACTGGCTGatgtctcttatt                                               >  1:166230/1‑290 (MQ=255)`  
                                                                                                                                                                                                                                                                     `aagaGGGGAAGAATAGTCAAACTCAGAGTCAACAGACTGAGCCAACGACACTGACTTGTGACAAGATTCTTGATGACTTTCCAGACGACGACGAGAAGTTTCGCTGGAGAGTCCCATTATAATACGACTCACTAAAGGAGACACACCATGTTCCAACTGATTAAGAAGTTAGACCAACTGCTGGTTCGTATGTACAACGTGGAAGCCAAGCGACTGAGCGATGAGTCTCGTAAAGAGGCCACACAGTCACGCGCTCTGGCGATTCGCTCCAACGAACTGGCTGACAGTGCATCCACTAAAg                                         <  1:211212/298‑1 (MQ=255)`  
                                                                                                                                                                                                                                                                     `aaatcgaaaggaatggTCACACTAAGAGTCAACAGACTGGTCCAACGACACTGACTTCTGACAGGATTCTTGATTACTTTCCAGACGACTACGAGAAGTTTCGCTGGAGAGTCCCATTCTAATACGACTCACTAAAGGAGACACACCATGTTCAAACTGATTAAGAAGTTAGGCCAACTGCTGGTTCGTATGTACAACGTGGAAGCCAAGCGACTGAACGATGAGGCTCGTAAAGAGGCCACACAGTCACGCGCTCTGGCGATTCGCTCCAACGAACTGGCTGACAGTGCATCCACTAAAg                                         <  1:69290/286‑1 (MQ=255)`  
                                                                                                                                                                                                                                                                         `ggggAAGAAGAGTCACACTCAGAGTCAACAGACTGGTCCAACGACACTGACTTCTGACAGGATTCTTGATGACTTTCCAGACGACTACGAGAAGTTTCGCTGGAGAGTCCCATTCTAATACGACTCACTAAAGGAGACACACCATGTTCAAACTGATTAAGAAGTTAGGCCAACTGCTGGTTCGTATGTACAACGTGGAAGCCAAGCGACTGAACGATGAGGCTCGTAAAGAGGCCACACAGTCACGCGCTCTGGCGATTCGCTCCAACGAACTGGCTGACAGTGCATCCACTAAAGtctg                                     >  1:205733/1‑298 (MQ=255)`  
                                                                                                                                                                                                                                                                           `gggagatgtgtataagagacagTCAACAGACTTGTCCAACGACACTGACTTCTGACAGGATTCTTGATGACTTTCCAGACGACTACGAGAAGTTTCGCTGGAGAGTCCCATTCTAATACGACTCACTAAAGGAGACACACCATGTTCAAACTGATTAAGAAGTTAGGCCAACTGCTGGTTCGTATGTACAACGTGGAAGCCAAGCGACTGAACGATGAGGCTCGTAAAGAGGCCACACAGTCACGCGCTCTGGCGATTCGCTCCAACGAACTGGCTGACAGTGCATCCACTAAAGTTACCg                                   <  1:384473/281‑1 (MQ=255)`  
                                                                                                                                                                                                                                                                            `gagatgtgtataagagacaggtcaacagactggtccaacgaCACTGACTTCTGACAGGATTCTTGAcagttgtttcatatgaagagattgttaagtcacgataatcaataggagaaatcaatatgatcgtttctgacatcgaagcaaacgccctcttagagagcgtcactaagttccactgcggggttatctacgactactccaccgctgagtacgtaagctaccgtccgagtgacttcggtgcgtatctggatgcgctggaagccgaggttgcacgaggcggtcttattgtgttccacaa                                  <  1:214308‑M1/281‑236 (MQ=255)`  
                                                                                                                                                                                                                                                                            `gaagaaGAGTCACACTCAGAGTCAACAGACTGGTCCAACGACACTGACTTCCGACAGGATTCTTGATGACTTTCCCGACGACTACGAGAAGTTTCGCTGGAGAGTCCCATTCTAATACGACTCACTAAAGGAGACACACCATGTTCCAACTGATTACGAAGTTAGGCCAACTGCTGGTTCGTATGTACAACGTGTAAGCCAACCTACTGAACGATGAGGCTCGTAAAGAGGCCTCACAGTCACGCGCTCTGGCGATTCCCTCCACCGAACTGGCTGACAGTGCATCCACCaacgcatgtct                                  >  1:290217/1‑292 (MQ=255)`  
                                                                                                                                                                                                                                                                                      `cacaCTCAGAGTCAACAGACTGGTCCAACGGCACTGACTTCTGACAGGATTCTTGAcagttgtttcatatgaagagattgttaagtcacgataatcaataggagaaatcaatatgatcgtttctggcatcgaagctaacgccctcttagagagcgtcactaagttccactgcggggttatctacgactactccaccgctgagtacgtaagctaccgtccgagtgacttcggtgcgtatctggatgcgctggaagccgaggttgcacgaggcggtcttattgtgttccacaacggtcacaag                        <  1:45193‑M1/301‑246 (MQ=255)`  
                                                                                                                                                                                                                                                                                       `aagacaggagtcaacagACTGGTCCAACGACACTGACTTCTGACAGGATTCTTGAcagttgtttcatatgaagagattgttaagtcacgataatcaataggagaaatcaatatgatcgtttctgacatcgaagctaacgccctcttagagagcgtcactaagttccactgcggggttatctacgactactccacagctgagtacgtaagctaccgtccgagtgacttcggtgcgtatctggatgcgctggaagccgaggttgcacgaggcggtcttattgtgttccacaacggtcacaagt                       <  1:367422‑M1/294‑247 (MQ=255)`  
                                                                                                                                                                                                                                                                                          `cTCAGAGTCAACAGACTGGTCCAACGACACTGACTTCTGACAGGATTCTTGAcagttgtttcatatgaagagattgttaagtcacgataatcaataggagaaatcaatatgatagtttctgacatcgaagctaacgccctcttagagagcgtcactaagttccactgcggggttatcaacgactactccaccgctgagtacgtaagctaccgtccgagtgacttcggtgcgtatatggatgcgctggaagccgaggttgcacgaggcggtcttattgtgttcaacaacggtcaaaagtatg                    >  1:126376‑M1/1‑52 (MQ=255)`  
                                                                                                                                                                                                                                                                                              `gagTCAACAGACTGGTCCAACGACACTGACTTCTGACAGGATTTTTGATGACTTTCCAGACGACTACGAGAAGTTTCGCTGGAGAGTCCCATTCTAATACGACTCACTAAAGGAGACACACCATGTTCAAACTGCTTAAGAAGTTCGGCCAACTGCTGGTTCGTATGTACAACGTGGAAGCCAAGCGACTGAACGATGAGGCTCGTAAAGAGGCCACACAGTCCCGCGCCCCGCCGATTCGCTCCAACGAACTGGCTGACAGTGCATCCACTAAAGTTACCGCGGCTGCCCGGGTTGCaca                >  1:354625/1‑299 (MQ=255)`  
                                                                                                                                                                                                                                                                                              `gagTCAACAGACTGGTCCAACGACACTGACTTCTGACAGGATTCTTGATGACTTTCCAGACGACTACGAGAAGTTTCGCTGGAGAGTCCCATTCTAATACGACTCACTAAAGGAGACACACCATGTTCAAACTGATTAAGAAGTTAGGCCAACTGCTGGTTCGTATGTACAACGTGGAAGCCAAGCGACTGAACGATGAGGCTCGTAAAGAGGCCACACAGTCACGCGCTCTGGCGATTCGCTCCAAAGAACTGGCTGACAGTGCATCCACTAAAGTTACCGAGGCTGCCCGTGTGGCaaa                >  1:168660/1‑301 (MQ=255)`  
                                                                                                                                                                                                                                                                                                `gTCAACATACTGGTCCAACGACACTGACTTCTGACAGGGTTCTTGATGACTTTCCAGACTACTACGAGAAGTTTCGCTGGATGTTCCCATTCTAATACGACTCACTAAAGGAGACACACCATGTTCAAACTGATTAAGAAGTTAGGCCAACTGCTGGTTCGAATGTACAACGTGGAAGCTAAGCGACTGAACGATGATGCTCGTAAATAGGCCACACAGTCACGCTCACTGGCGATTCGCTCCAACGAACTTGCTGACAGTTCATCCACTACAGTTACCGAGGCTTCCCGTGTGGcgaccc              >  1:190686/1‑296 (MQ=255)`  
                                                                                                                                                                                                                                                                                                 `tCAACAGACTGGTCCAACGACACTGACTTCTGACAGGATTCTTGATGACTTTCCAGACGACTACGAGAAGTTTCGCTGGAGAGTCCCATTCTAATACGACTCACTAAAGGAGACACACCATGTTCAAACTGATTAAGAAGTTAGGCCAACTGCTGGTTCGTATGTACAACGTGGAAGCCAAGCGACTGAACGATGAGGCTCGTAAAGAGGCCACACAGTCACGCGCTCTGGCGATTCGCTCCAACGAACTGGCTGACAGTGCATCCACTAAAGTTACCGAGGCTGCCCGTGTGGCAAACCa             <  1:26852/301‑1 (MQ=255)`  
                                                                                                                                                                                                                                                                                                  `cAACAGACTGGTCCAACGACACTGACTTCTGACAGGATTCTTGATGACTTTCCAGACGACTACGAGAAGTTTCGCTCGAGAGTCCCATTCTAATACGACTCACTAAAGGAGACACACCATGTTCCAACTGATTAAGAAGTTATGCCAACTGCTGGTTCGTATGTACAACGTGGAAGCCAAGCTACTGAACGCTGAGGCTTGTAAACAGGCCACACAGTCACTCGCTCTGGCGATTCGCTCCAACGAACTGGCTGTCAGTGCATCCACTAAAGTTCCCGAGGCTGCCCGTGTGGCAAACCaa            >  1:129754/1‑301 (MQ=255)`  
                                                                                                                                                                                                                                                                                                      `aaacaggtccaaCGACACTGACTTCTGACAGGATTCTTGAcagttgtttcatatgaagagattgttaagtcacgataatcaataggagaaatcaatatgatcgtttctgacatcgaagctaacgccctcttagagagcgtcactacgttccactgcggggttatctacgactactccaccgctgagtacgtaagctaccgtcagagtgacttcggtgcgtatctggctgcgctggaagccgaggttgcacgaggcggtcttattgtgttccacaacggtcacaagtatgacgttcctgcat        <  1:77448‑M1/296‑262 (MQ=255)`  
                                                                                                                                                                                                                                                                                                      `aGACTTGTCCAACGACAATGACTTCTGACAGGATTCTTGAcagttgtttcatatgaagagattgttaagtcacgataatcaataggagaaatcaatatgatcgtttctgacatcgaagctaacgccctcttagagagcgtcactaagttccactgcggggttatctacgactactccaccgctgagtacgtaagctaccgtccgagtgacttcggtgcgtatctggatgcgctggaagccgaggttgcacgaggcggtcttattgtgttccacaacggtcacaagtatgacgttactgcat        <  1:504430‑M1/301‑262 (MQ=255)`  
                                                                                                                                                                                                                                                                                                      `aGACTGGTCCAACGACACGGACTTCTGACAGGATTCTTGAcagttgtttcatatgaagagattgttaagtcacgataatcaataggagaaataaatatgatcgtttctgacatcgaagctaacgccctcttagagagcgtcactaagttccactgcggggttatctacgactactccaccgctgagtacgtaagctaccgtccgagtgacttcggtgcgtatctggatgcgctggaagccgaggttgcacgaggcggtcttattgtgttccacaacggtcacaagtatgacgttcctgcat        <  1:468889‑M1/301‑262 (MQ=255)`  
                                                                                                                                                                                                                                                                                                       `gACTGGTCCAACGACACTGACTTCTGACAGGATTCTTGATGACTTTCCAGACGACTACGAGAAGTTTCGCTGGAGAGTCCCATTCTAATACGACTCACTAAAGGAGACACACCACGTTCAAACTGATTAAGAAGTCAGGCTAACTGTTGGTTCGTATGTACAACGTGGAAGCCAAGCGACTGAACGATGAGGTCCGTAAAGAGGCCACACAGTCACGCGTCCTGGCGATTCGCTCCAACGAACTGGCTGACAGTGCATCCACTAAATTTACCGAGGCTGCCCGTGTGGCAAACCACACTCa       >  1:32593/1‑301 (MQ=255)`  

                                                                                                                                                                                                                                                                                                            `|`                                                                                                                                                                                                                                                                                                              
`GGTCGTAATTTGTCACCTTAAGAACCCAGACAAAGGTAAAGCACATGAGGAAGGTCGCCCCGTTTCTATTACTGACCTACGTGGTTCTGGCGCACTACGCCAACTATCTGATACTATTATTGCCCTTGAGCGTAATCAGCAAGGCGATATGCCTAACCTTGTCCTCGTTCGTATTCTCAAGTGCCGCTTTACTGGTGATACTGGTATCGCTGGCTACATGGAATACAACAAGGAAACCGGATGGCTTGAACCATCAAGTTACTCAGGGGAAGAAGAGTCACACTCAGAGTCAACAGACTGGTCCAACGACACTGACTTCTGACAGGATTCTTGATGACTTTCCAGACGACTACGAGAAGTTTCGCTGGAGAGTCCCATTCTAATACGACTCACTAAAGGAGACACACCATGTTCAAACTGATTAAGAAGTTAGGCCAACTGCTGGTTCGTATGTACAACGTGGAAGCCAAGCGACTGAACGATGAGGCTCGTAAAGAGGCCACACAGTCACGCGCTCTGGCGATTCGCTCCAACGAACTGGCTGACAGTGCATCCACTAAAGTTACCGAGGCTGCCCGTGTGGCAAACCAAGCTCAACAGC  >  NC_001604.1/12944‑13544`

  

Alignment Legend

Aligned base mismatch/match (shaded by quality score): `ATCG/ATCG` `< 3 ≤` `ATCG/ATCG` `< 10 ≤` `ATCG/ATCG` `< 18 ≤` `ATCG/ATCG` `< 32 ≤` `ATCG/ATCG` `< 38 ≤` `ATCG/ATCG`

Unaligned base: `atcg`    Masked matching base: `atcg`    Alignment gap: `‑`    Deleted base: `‑`

Reads not counted as support for junction

read\_name Not counted due to insufficient overlap past the breakpoint.

read\_name Not counted due to not crossing MOB target site duplication.
